# Supplementary material for: Visualization of conformational changes and membrane remodeling leading to genome delivery by viral class-II fusion machinery
Source: Nat Commun. 2022 Aug 15;13:4772. doi: 10.1038/s41467-022-32431-9 (PMC9378758; doi:10.1038/s41467-022-32431-9)
Supplement: Supplementary file 1 — Supplementary Information [file 41467_2022_32431_MOESM1_ESM.pdf]

Supplementary Information:

Visualization of conformational changes and membrane remodeling leading to genome delivery by viral class-II fusion machinery

Vidya Mangala Prasad[1¥], Jelle S. Blijleven[2], Jolanda M. Smit[3], Kelly K. Lee[1,4,5*]

Affiliations:

1. Department of Medicinal Chemistry, University of Washington, Seattle, WA 98195, USA.
2. Biological Physics, Structure and Design Graduate Program, University of Washington, Seattle, WA 98195, USA.
3. Department of Microbiology, University of Washington, Seattle, WA 98195, USA.
4. Zernike Institute for Advanced Materials, University of Groningen, Groningen, The Netherlands.
5. Department of Medical Microbiology and Infection Prevention, University of Groningen, University Medical Center Groningen, Groningen, The Netherlands.

¥Current Address: Molecular Biophysics Unit, Indian Institute of Science, Bengaluru 560012, Karnataka, India.

*Correspondence to: kklee@uw.edu

Supplementary Figures:

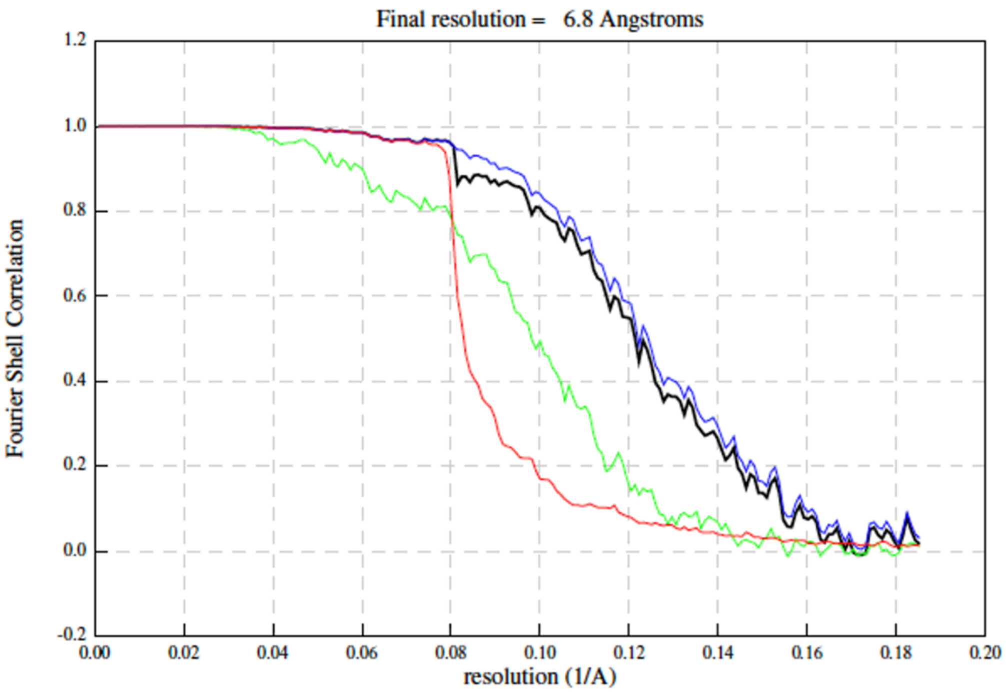

Supplementary Figure 1: Gold-standard Fourier shell correlation (FSC) curves. Lines represent correlation corrected (black), masked (blue), unmasked (green), and phase randomized (red) Fourier Shell correlation curve maps. Resolution at "gold-standard" FSC cutoff of 0.143 is given as final resolution.

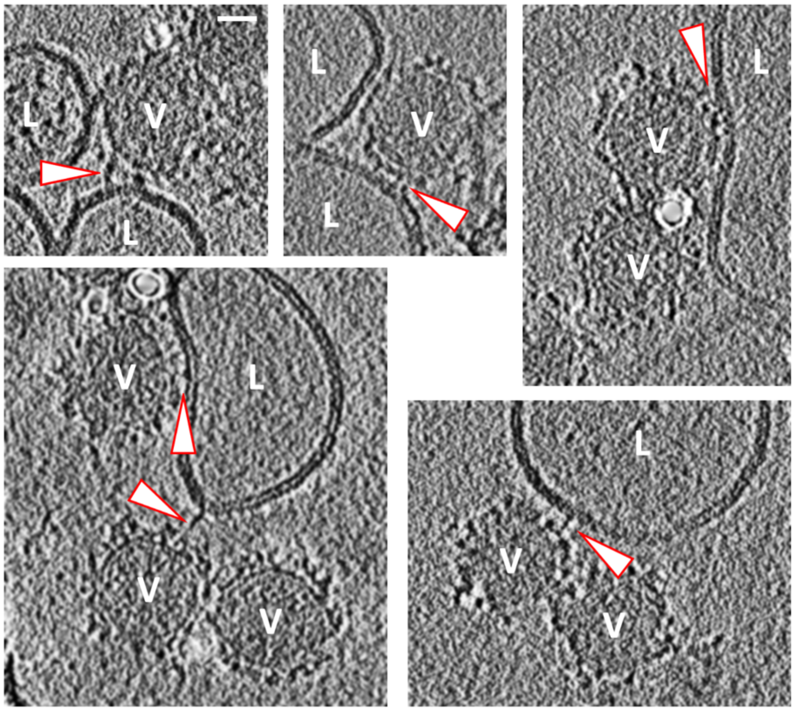

Supplementary Figure 2: CHIKV-liposome interactions at pH >6.0. Discrete densities (arrow heads) connecting CHIKV (V) to surrounding liposomes (L) is seen at pH 6.3 (top panels) and pH 6.1 (bottom panels). Total of n=14 independent events were observed for pH 6.3 and pH 6.1 together. Black is high density. Scale bar is 200 Å in length.

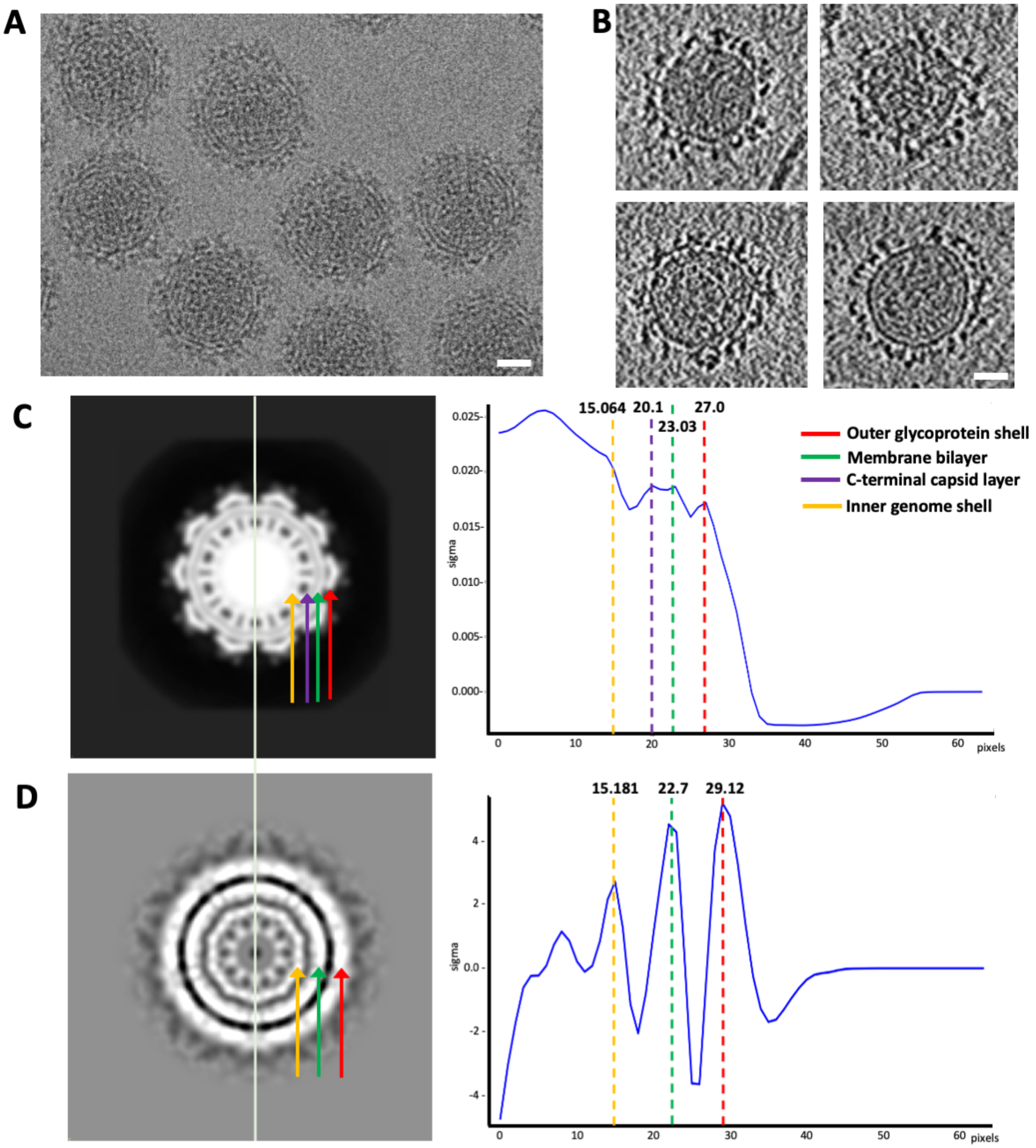

Supplementary Figure 3: Sub-tomogram averaging analysis of low pH (<6.0) treated CHIKV. A. EM micrograph of neutral pH CHIKV. Representative micrograph from total of 495 independent images collected. B. Tomogram cross-sections of low pH treated CHIKV. Representative virions shown from a total of n=70 sub-tomograms. In both panels A and B, black

is high density and scale bar is 200 Å. C. Left: Cross-section of neutral pH CHIKV EM density map low pass filtered to 45Å resolution. Right: 2D radial density plot of the same. D. Left: Cross-section of low pH treated CHIKV sub-tomogram averaged EM density map at 45Å resolution showing lack of discernable protein features (as compared to panel C). Right: 2D radial density plot of the same. In both panels C and D, red arrow or dotted line indicates the outer glycoprotein shell, green arrow or dotted line indicates the membrane bilayer, purple arrow or dotted line indicates the nucleocapsid shell juxtaposed underneath the viral membrane, yellow arrow or dotted line indicates the outer radius of the central genome region. The 2D radial density plot shows that though the internal genome region and membrane bilayer are at similar radii, the glycoprotein shell peak is at a marginally higher radius in the low pH treated virion when compared to the neutral pH CHIKV. Pixel size of density maps and related plots are 10.14 Å/pixel.

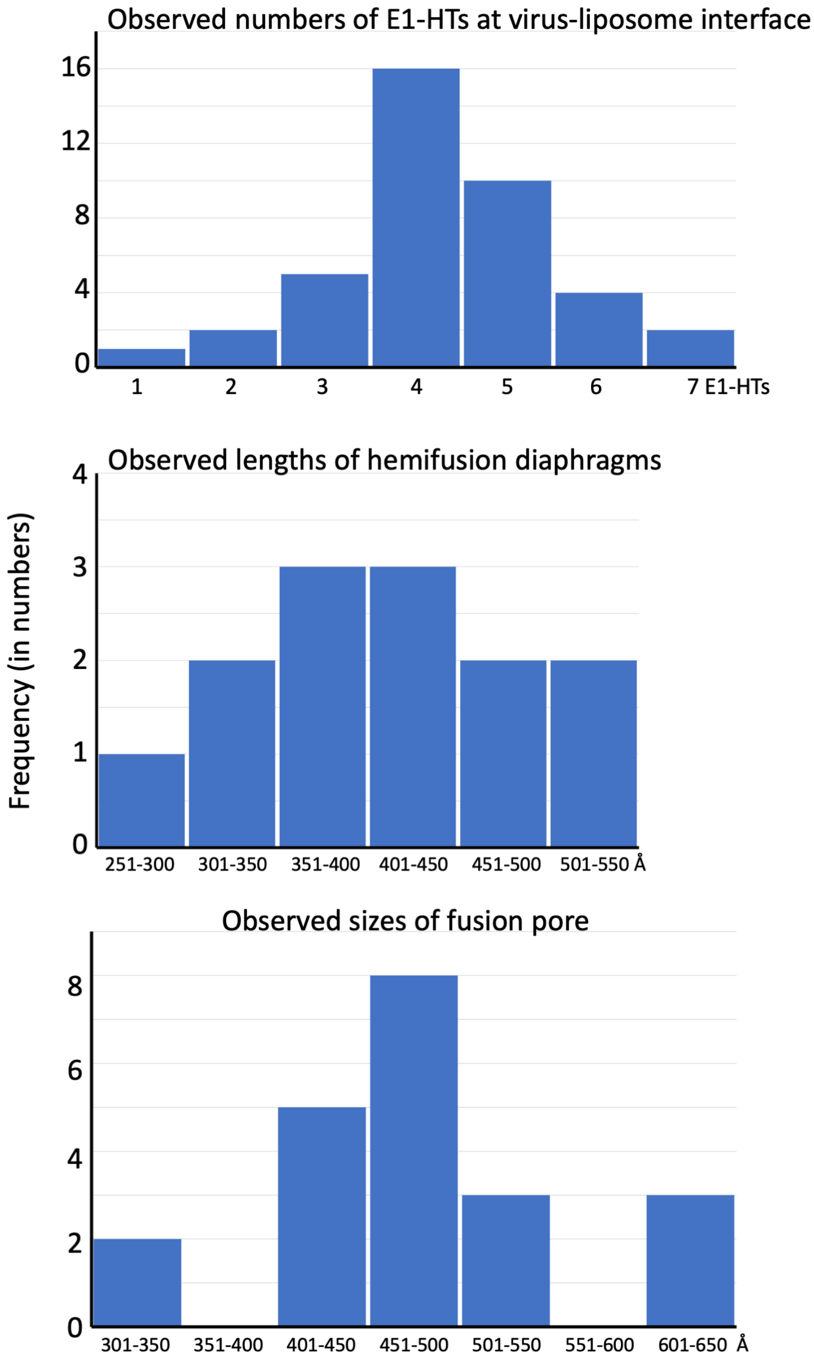

Supplementary Figure 4. Distribution of intermediate states. Top panel: Frequency of number of E1-HTs observed at a given virus-liposome interface (n=40 virus-liposome interfaces from independent tomograms). Middle panel: Distribution of observed hemifusion diaphragm sizes (n=13 hemifusion interfaces from independent tomograms). Bottom panel: Distribution of fusion pore sizes observed in cryo-ET data (n=21 fusion pores from independent tomograms). Source data are available as a Source Data file.

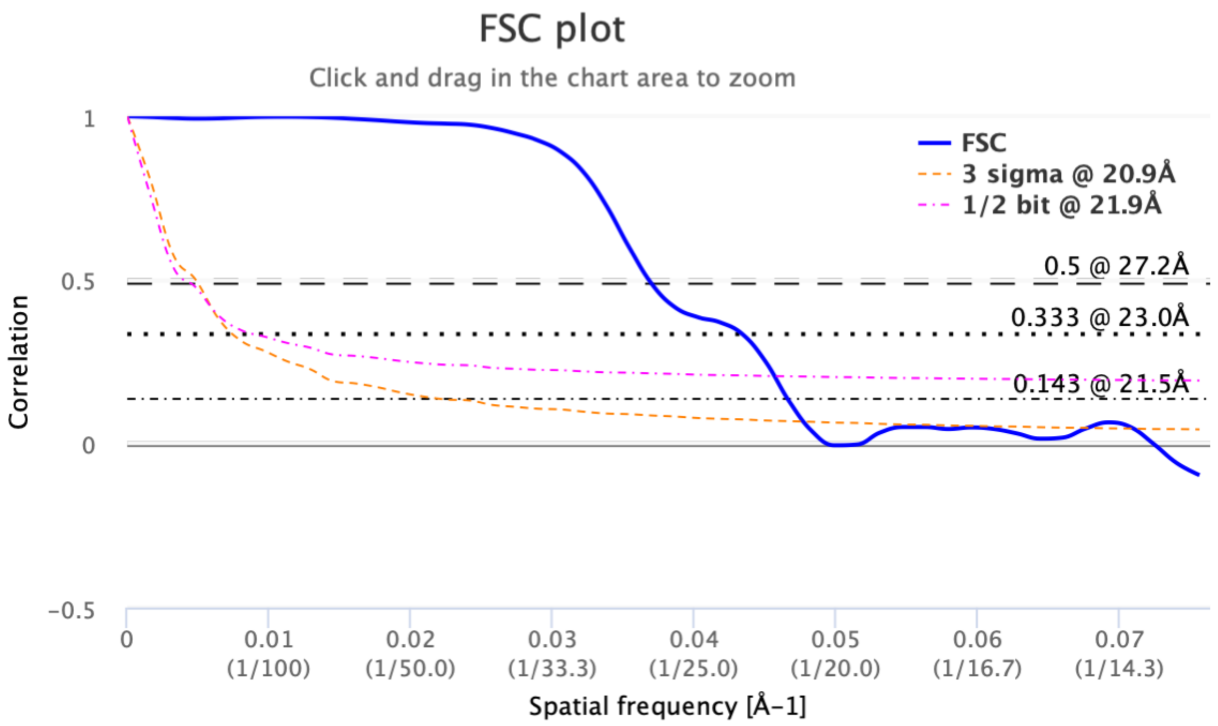

Supplementary Figure 5: Fourier Shell correlation (FSC) curve. FSC curve computed using even and odd half-maps calculated for the sub-tomogram averaged post-fusion E1 trimers. Plot was calculated using the Electron Microscopy DataBank (EMDB) FSC server.

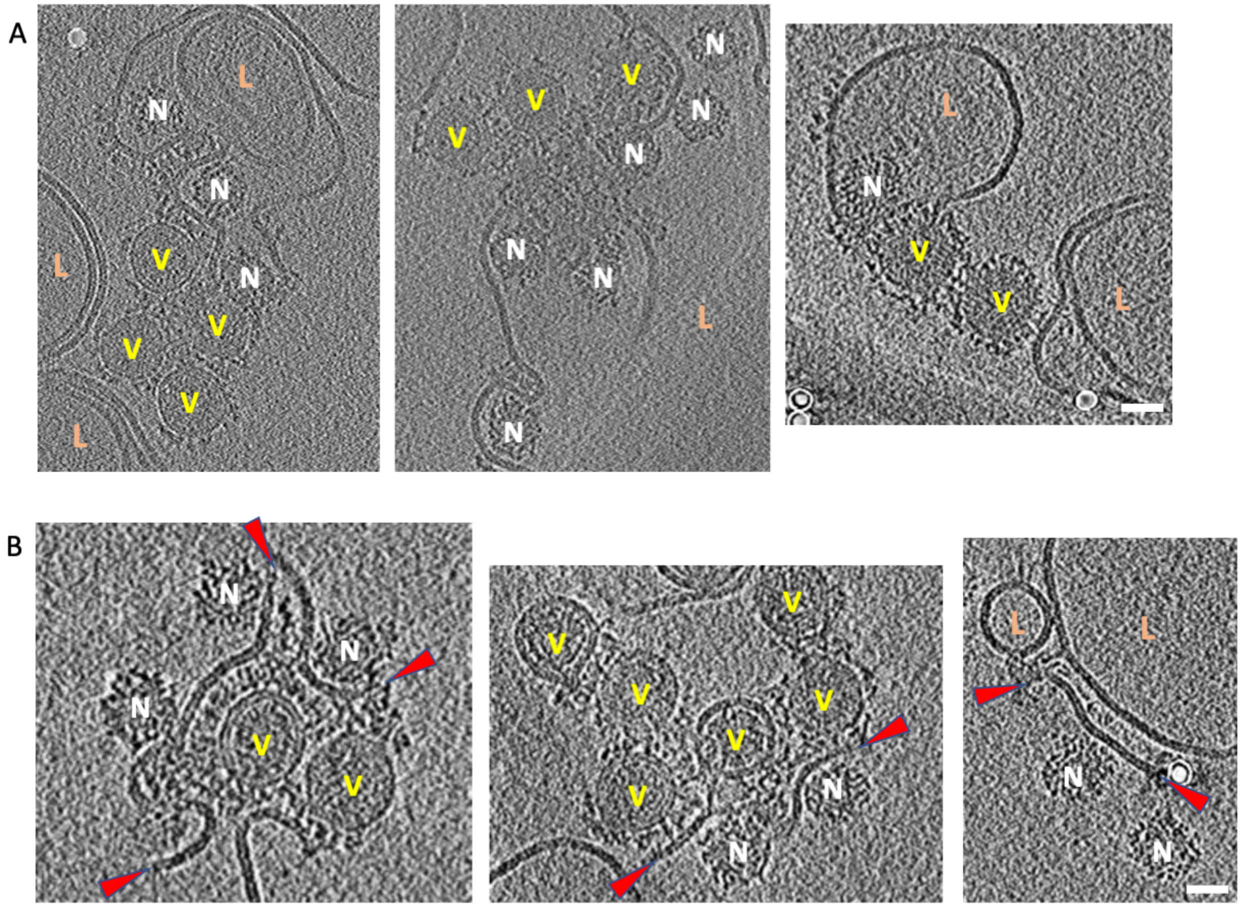

Supplementary Figure 6: Effect of prolonged exposure at low pH (pH 5.6 and pH 5.1) on CHIKV. A. Representative tomogram cross-sections showing examples of multiple CHIKV aggregates fusing with liposomes (Total of n=31 such independent events were observed in our dataset). B. Representative tomogram cross-sections showing examples of CHIKV fusing with other virions and releasing its nucleocapsids (Total of n=16 such independent events were observed in our dataset). Red arrowheads indicate the ends of the fused virus membranes. In all panels: Black is high density. Virus is denoted as V, liposomes as L and nucleocapsids as N. Scale bars are 300Å in length.

Supplementary Table 1: Cryo-EM data collection, refinement and validation statistics

	Single particle cryo-EM map of neutral pH CHIKV (S27) (EMDB-27559)	Sub-tomogram averaged structure of post-fusion full-length E1 glycoprotein (EMDB-27248) (PDB 8D87)
**Data collection and processing**		
Magnification	105000X	81000X
Voltage (kV)	300	300
Electron exposure (e–/Å^2)	43.89	~60-80
Defocus range (μm)	-1.5 to -3.5	-2.5 to 5
Pixel size (Å)	1.35	1.69
Symmetry imposed	Icosahedral	C3
Initial particle images (no.)	7741	591
Final particle images (no.)	5806	591
Map resolution (Å) FSC threshold	6.75 (0.143 FSC)	27.2 (0.5 FSC)
Map resolution range (Å)	-	-
**Refinement**		
Initial model used (PDB code)		1RER (Rigid body fit)
