# Supplementary material for: Visualization of conformational changes and membrane remodeling leading to genome delivery by viral class-II fusion machinery
Source: Nat Commun. 2022 Aug 15;13:4772. doi: 10.1038/s41467-022-32431-9 (PMC9378758; doi:10.1038/s41467-022-32431-9)
Supplement: Supplementary file 2 — Description of Additional Supplementary Files [file 41467_2022_32431_MOESM2_ESM.pdf]

Description of Additional Supplementary Files

File Name: Supplementary Movie 1
Description: **Stage Imembrane recruitment.** Cryoelectron tomography reconstruction of CHIKV-liposome interaction showing an example of stage I – membrane recruitment. CHIKV is indicated as V, liposome as L and interaction site is indicated by a yellow arrow. Scale bar is 200 Å. Black is high density. For all supplemental movies, tomogram pixel size in direction perpedicular to electon beam (x-y direction) is 10.14 Å/pixel and 50.7 Å/pixel in the direction of the electron beam (zdirection).

File Name: Supplementary Movie 2
Description: **Stage IImembrane attachment.** Cryoelectron tomography reconstruction of CHIKV-liposome interaction showing an example of stage II – membrane attachment. CHIKV is indicated as V, liposome as L and interaction site is indicated by yellow arrows. Green arrow indicates the presence of a stage III – E1-HT example in the same virion. Scale bar is 200 Å. Black is high density.

File Name: Supplementary Movie 3
Description: **Stage IIIE1-HT formation.** Cryo-electron tomography reconstruction of CHIKV-liposome interaction showing an example of stage III – formation of E1-HT. CHIKV is indicated as V, liposome as L and interaction site is indicated by yellow arrow. Green arrow indicates the presence of a stage IV – E1-HT membrane insertion example in an adjacent virion. Scale bar is 200 Å. Black is high density.

File Name: Supplementary Movie 4
Description: **Stage IVE1-HT membrane insertion.** Cryoelectron tomography reconstruction of CHIKV-liposome interaction showing an example of stage IV –E1- HT membrane insertion. CHIKV is indicated as V, liposome as L and interaction site is indicated by yellow arrow. Scale bar is 200 Å. Black is high density.

File Name: Supplementary Movie 5
Description: **Stage Vopposing membrane superposition.** Cryo-electron tomography reconstruction of CHIKV-liposome interaction showing an example of stage V – opposing membrane superposition. CHIKV is indicated as V, liposome as L and interaction site is indicated by yellow arrow. Scale bar is 200 Å. Black is high density.

File Name: Supplementary Movie 6
Description: **Stage VItight membrane apposition.** Cryoelectron tomography reconstruction of CHIKV-liposome interaction showing an example of stage VI – tight membrane apposition. CHIKV is indicated as V, liposome as L and interaction site is indicated by yellow arrow. Scale bar is 200 Å. Black is high density.

File Name: Supplementary Movie 7
Description: **Stage VII-hemifusion.** Cryo-electron tomography reconstruction of CHIKV-liposome interaction showing an example of stage VII – hemifusion. CHIKV is indicated as V and liposome as L. Hemifusion site is indicated by yellow arrows. Scale bar is 200 Å. Black is high density.

File Name: Supplementary Movie 8
Description: **Stage VIII-fusion pore formation.** Cryoelectron tomography reconstruction of CHIKV-liposome interaction showing an example of stage VIII – fusion pore formation. CHIKV is indicated as V, liposome as L and interaction site is indicated by yellow arrow. Scale bar is 200 Å. Black is high density.

File Name: Supplementary Movie 9

Description: **Stage IXnucleocapsid release.** Cryo-electron tomography reconstruction of CHIKV-liposome interaction showing an example of stage IX – nucleocapsid release. Liposome is labeled as L. Yellow arrows indicate two post-fusion nucleocapsids released into the liposome lumen. Green arrows denote post-fusion E1 glycoprotein trimers distributed on the liposome membrane. Scale bar is 200 Å. Black is high density.
