# Supplementary material for: Visualization of conformational changes and membrane remodeling leading to genome delivery by viral class-II fusion machinery
Source: Nat Commun. 2022 Aug 15;13:4772. doi: 10.1038/s41467-022-32431-9 (PMC9378758; doi:10.1038/s41467-022-32431-9)
Supplement: Supplementary file 12 — Reporting Summary [file 41467_2022_32431_MOESM12_ESM.pdf]

Corresponding author(s): Dr. Kelly K. Lee

Last updated by author(s): 2022/07/6

Reporting Summary

Nature Portfolio wishes to improve the reproducibility of the work that we publish. This form provides structure for consistency and transparency in reporting. For further information on Nature Portfolio policies, see our Editorial Policies and the Editorial Policy Checklist.

Statistics

For all statistical analyses, confirm that the following items are present in the figure legend, table legend, main text, or Methods section.

n/a	Confirmed	
☐	☒	The exact sample size (*n*) for each experimental group/condition, given as a discrete number and unit of measurement
☐	☒	A statement on whether measurements were taken from distinct samples or whether the same sample was measured repeatedly
☒	☐	The statistical test(s) used AND whether they are one- or two-sided<br>*Only common tests should be described solely by name; describe more complex techniques in the Methods section.*
☒	☐	A description of all covariates tested
☒	☐	A description of any assumptions or corrections, such as tests of normality and adjustment for multiple comparisons
☐	☒	A full description of the statistical parameters including central tendency (e.g. means) or other basic estimates (e.g. regression coefficient) AND variation (e.g. standard deviation) or associated estimates of uncertainty (e.g. confidence intervals)
☒	☐	For null hypothesis testing, the test statistic (e.g. *F*, *t*, *r*) with confidence intervals, effect sizes, degrees of freedom and *P* value noted<br>*Give P values as exact values whenever suitable.*
☒	☐	For Bayesian analysis, information on the choice of priors and Markov chain Monte Carlo settings
☒	☐	For hierarchical and complex designs, identification of the appropriate level for tests and full reporting of outcomes
☒	☐	Estimates of effect sizes (e.g. Cohen's *d*, Pearson's *r*), indicating how they were calculated

Our web collection on statistics for biologists contains articles on many of the points above.

Software and code

Policy information about availability of computer code

Data collection	Leginon, SerialEM
Data analysis	MotionCor2, IMOD 4.10, PEET 1.12.0, ImageJ v1.51, UCSF Chimera, Biorender

For manuscripts utilizing custom algorithms or software that are central to the research but not yet described in published literature, software must be made available to editors and reviewers. We strongly encourage code deposition in a community repository (e.g. GitHub). See the Nature Portfolio guidelines for submitting code & software for further information.

Data

Policy information about availability of data

All manuscripts must include a data availability statement. This statement should provide the following information, where applicable:

- Accession codes, unique identifiers, or web links for publicly available datasets
- A description of any restrictions on data availability
- For clinical datasets or third party data, please ensure that the statement adheres to our policy

Sub-tomogram averaged density map of post-fusion E1 trimer with corresponding fitted atomic model has been deposited with accession codes EMD-27248 and PDB ID 8D87. Single particle cryo-EM map of Chikungunya virus at neutral pH is deposited with accession code EMD-27559.

Human research participants

Policy information about studies involving human research participants and Sex and Gender in Research.

Reporting on sex and gender	NA
Population characteristics	NA
Recruitment	NA
Ethics oversight	NA

Note that full information on the approval of the study protocol must also be provided in the manuscript.

Field-specific reporting

Please select the one below that is the best fit for your research. If you are not sure, read the appropriate sections before making your selection.

- [x] Life sciences
- [ ] Behavioural & social sciences
- [ ] Ecological, evolutionary & environmental sciences

For a reference copy of the document with all sections, see nature.com/documents/nr-reporting-summary-flat.pdf

Life sciences study design

All studies must disclose on these points even when the disclosure is negative.

Sample size	Sample sizes where determined by counting the number of cryo-electron tomograms generated and the number of virus-liposome complexes observed in the tomograms. No statistical methods were involved.
Data exclusions	No data was excluded from analyses
Replication	Multiple data collections were done at given experimental conditions (as presented in the manuscript) to verify the reproducibility of the results. Each experiment was performed independently a minimum of 3 times
Randomization	No statistical calculation was involved that require randomization
Blinding	There were no control groups involved in the study and did not need blinding as these are molecular experiments.

Reporting for specific materials, systems and methods

We require information from authors about some types of materials, experimental systems and methods used in many studies. Here, indicate whether each material, system or method listed is relevant to your study. If you are not sure if a list item applies to your research, read the appropriate section before selecting a response.

Materials & experimental systems

n/a	Involved in the study	
x		Antibodies
	x	Eukaryotic cell lines
x		Palaeontology and archaeology
x		Animals and other organisms
x		Clinical data
x		Dual use research of concern

Methods

n/a	Involved in the study	
x		ChIP-seq
x		Flow cytometry
x		MRI-based neuroimaging

Eukaryotic cell lines

Policy information about cell lines and Sex and Gender in Research

Cell line source(s)	BHK-21 (Baby Hamster Kidney Cells) (ATCC)
Authentication	None
Mycoplasma contamination	Negative
Commonly misidentified lines (See ICLAC register)	No commonly misidentified lines were used in the study.
